# Perceived bloggers’ competence as a moderator of the relationship between information quality, source credibility, and health information adoption in social media

**DOI:** 10.1371/journal.pone.0348197

**Published:** 2026-06-01

**Authors:** Changning Ren, Akmar Hayati Ahmad Ghazali, Syafila Kamarudin, Xuemei Chen, Ye He

**Affiliations:** Faculty of Modern Language and Modern Communication, Universiti Putra Malaysia, Selangor Darul Ehsan, Malaysia; Institute of Medical Biochemistry Leopoldo de Meis (IBqM) - Federal University of Rio de Janeiro (UFRJ), BRAZIL

## Abstract

**Backgroud:**

In the context of digital transformation, social media has become a key channel for the public to obtain health information, and bloggers play an important role in shaping public health behaviors. However, the internal mechanism of how PerceivedBloggers’ Competence (PBC) and information characteristics jointly affect Information Adoption (IA) has not been fully revealed.

**Aim:**

This study integrates the Information Adoption Model (IAM) with Social Cognitive Theory (SCT) to examine the relationship between Perceived Bloggers’ Competence and Information Adoption, and to explore how Perceived Bloggers’ Competence moderates the impact of Information Quality (IQ) and Information Credibility (IC) on information adoption through Information Usefulness (IU).

**Method:**

A total of 1219 participants (648 males and 571 females) were recruited using a cross-sectional online survey design. Data were collected through a structured questionnaire and analyzed using SPSS and AMOS. This study constructs a conceptual model with Information Usefulness as a mediator and perceived bloggers’ competence as a moderating variable and employs path analysis along with moderating effect tests to verify the hypotheses.

**Results:**

In the study, Information Quality and Information Credibility had significantly positive effects on Information Usefulness, which in turn strongly predicted Information Adoption, also Perceived Bloggers’ Competence negatively moderates the relationship between information characteristics and adoption.

**Conclusion:**

This study challenges the assumption that bloggers’ expertise necessarily promotes Information Adoption, also provides key insights for health communicators, showing that striking a balance between expertise and accessibility is essential for effective public health messaging on social media, exhibits an effect that can be called “competence discounting”.

## Introduction

The digital transformation of health communication has fundamentally reshaped the way of dissemination of public health information, and social networks are becoming the leading platforms for health-related guidance [1]. Recent statistics show that more than 70% of adults now turn to social media to access health information, highlighting their growing influence in shaping public perceptions and behaviors [2]. In their ever-changing landscape, bloggers— from medical professionals to lifestyle influencers—play a crucial role in guiding health behaviors. Their influence goes beyond content dissemination to shaping trust, engagement, and ultimately, health-related decision-making processes [3]. Despite increasing reliance on bloggers to provide health advice, the mechanism for the interaction of blogger attributes and information characteristics to affect adoption has not been fully explored. Previous research has mainly focused on content-related factors, such as information quality or source credibility, which are usually isolation [4]. This research gap is particularly important in the specific context of Malaysia. The country has a dual-track public-private health care system, and despite the high affordability of public health care, long waits often make the population, especially young people, vulnerable to daily health problems, prioritizing faster solutions. Therefore, self-treatment and pre-searching for health information on social media have become a common preventive measure [5]. However, this method ignores how audience’s perception of bloggers’ competence may shape the cognitive and emotional process behind information adoption (IA). Especially in the context of global health crises, which is characterized by institutional mistrust, political polarization, and an influx of unproven personal claims, it is crucial to understand how perception affects behavioral outcomes [6].

To bridge this gap, the present study integrates the Information Adoption Model (IAM) and Social Cognitive Theory (SCT) to investigate how perceived blogger competence (PBC) moderates the relationship between information characteristics (information quality and information credibility) and information adoption, mediated by information usefulness. By clarifying these approaches, this study helps advance the theoretical progress of digital health communication and provides practical strategies to reduce misinformation and promote evidence-based decision-making.

## Literature review

Since its independence in 1957, Malaysia’s health care system has undergone major organizations and revolutions. Its core development is a fundamental shift from traditional “disease treatment” to “medical services” focused on prevention, and its continued commitment to improving fairness and access to medical services [7]. Nevertheless, Malaysia still has a dual-track public-private health care system, and despite the high affordability of public health care, long waits often make people, especially young people, seeking faster solutions as a top priority [8]. Therefore, self-medical and pre-retrieval of health information on social media have become a common front-end behavior, which makes it of great practical significance to explore the adoption mechanism of social media health information in the local context [9].

The existing research on self-care in Malaysia shows a multi-angle development context. Earlier studies have focused on the prevalence of the practice, such as a survey of undergraduates in Penang found that 65.5% practiced self-medication in 2003 [9]. With the rise of social media, the research focus has been expanded to information access channels, pointing out that MySpace, Twitter, YouTube, Facebook and other platforms have become the easiest media for consumers to obtain health information [10]. Malaysia’s social media users show interest in the self-care content [11]. However, this convenience is also accompanied by risks, and the study also warns of misleading health information on social media [10]. In addition, personal factors such as education level are significantly related to the perception of self-care knowledge [12]. The COVID-19 pandemic has further changed the information environment. Many Malaysian undergraduate students are unusually active in obtaining information on social media after the pandemic [13].

The digital transformation in the field of health communication has fundamentally reshaped the pattern of public health information dissemination [14]. The core of this transformation is the Information Adoption Model (IAM), which assumes that high-quality information, defined as accuracy, relevance and consistency, directly enhances the information usefulness and eventually acts as a precursor to behavioral adoption [15,16]. Evidence supports this model; for instance, research shows that COVID-19 guidelines based on clinical evidence have achieved a higher adoption rate than guidelines based only on anecdotal claims [15]. These findings confirm the importance of information quality. However, the traditional IAM framework regards information quality as a static structure and ignores the dynamic role of intermediaries, specifically bloggers, in the social media ecosystems. In short, these studies paint a picture of self-medical behavior in Malaysia, which has a long history, high dependence on social media channels, a complex information environment and personal factors. Especially in the context of frequent global public health crises, declining institutional trust, political polarization, and an unverified surge in personal statements, an in-depth understanding of how perceived bloggers’ competence affects behavioral outcomes has been critical.

In practice, many users do not have the technical expertise required to independently evaluate complex medical or health-related content [17]. On the contrary, they rely on bloggers to distill and translate this information to form insights that are easier to understand and feasible [18]. This dependence shows that the impact of information quality on information usefulness is not an isolated phenomenon, but deeply embedded in the interaction between the information itself and the situational intermediaries provided by these online communicators [19]. Neurocognitive research further supports this view, showing that contact simplified interpretation can enhance the value calculation and decision-making process [20]. Considering these findings, we assume that:

H1: Information Quality (IQ) positively influences public health Information Usefulness (IU).

Similarly, the traditional application of the Information Adoption Model (IAM) has used fixed tags (such as professional titles and institutional affiliations) to realize the operationalization of information credibility [21]. However, taking into account the dynamic and interactive nature of social media, this static view does not seem to be sufficient. Social Cognitive Theory [22] provides a more detailed perspective, which suggests that credibility is not innate, but jointly constructed through real-time interaction and observable demonstrations of expertise. For instance, bloggers who actively expose misinformation in real time gain higher credibility than those who only rely on traditional professional knowledge tags [16]. This evidence indicates the need for a more flexible conceptualization of information credibility. Therefore, we propose:

H2: Information Credibility (IC) positively influences Information Usefulness (IU).

Although the Information Adoption Model (IAM) has advantages, its traditional use of information usefulness as a mediator is limited by its linear and static treatment of the relationship between the previous cause (information quality and information credibility) and information adoption. Empirical observations shows that the mediation process is much more complex and is significantly affected by situational moderators such as perceived bloggers’ competence [23]. User’s dependence on bloggers to explain complex information highlights that the mere presence of high-quality content does not guarantee its effective adoption, unless the information is appropriately placed in the situation. Neuroimaging evidence supports this argument; exposure to simplified and well-contextualized information can improve comprehension and promote more robust decision-making [20]. Therefore, we put forward the following mediating hypotheses:

H3: Information Usefulness (IU) mediates the effect of information Quality (IQ) on Information Adoption (IA).

H4: Information Usefulness (IU) mediates the effect of Information Credibility (IC) on Information Adoption (IA).

Regarding the moderating role of perceived blogger competence, it is crucial to realize that blogger competence is not just technical competence. It embodies the bloggers’ ability to interpret, contextualize, and convey complex information, which resonates with their audience and is easy to understand. Social Cognitive Theory emphasizes that such competence is vital in bridging the “competence discounting” [3]. Empirical data indicate that health-related advice provided by bloggers with high perceived competence, such as those who engaging in interactive Q&A sessions or use visual aids, is more likely to be adopted than static content presentations [24]. This suggests that the perceived bloggers’ competence not only strengthens the direct relationship between information usefulness and information adoption but also enhances the overall mediated pathways from information quality and information credibility to adoption. In contrast, the Internet is also a platform that is prone to false information, so the willingness and ability to share are particularly important [25]. Thus, we put forward the following moderating hypotheses:

H5: Perceived Blogger Competence (PBC) positively moderates the indirect effect of Information Quality (IQ) on Information Adoption (IA) via Information Usefulness (IU).

H6: Perceived Blogger Competence (PBC) positively moderates the indirect effect of Information Credibility (IC) on Information Adoption (IA) via Information Usefulness (IU).

**Fig 1:**
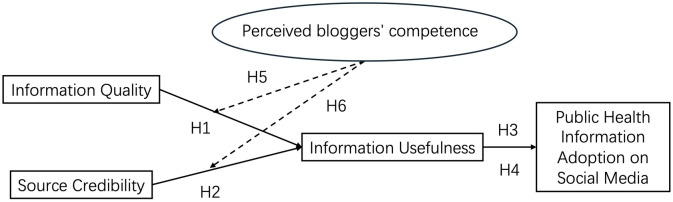
Conceptual Framework (N = 1219).

The key is that although the traditional Information Adoption Model (IAM) provides a robust framework for understanding information adoption, its limitation is that its fails to explain the dynamic interaction between static information attributes and active mediation provided by bloggers. Integrating Social Cognitive Theory into this framework can more comprehensively understand how trust and interactive participation transform information quality into practical behavioral outcomes. In the current era of widespread misinformation, the integration is particularly prominent because the costs of wrong health decision-making is huge in scale and global [26]. Thus, the hypotheses extend the theoretical basis of IAM and provide practical insights for optimizing health communication strategies in the digital environments. The following is the research model (Fig 1).

Based on the reviewed literature and theoretical framework, the following hypotheses are proposed:

H1: Information Quality (IQ) positively influences public health Information Usefulness (IU).

H2: Information Credibility (IC) positively influences Information Usefulness (IU).

H3: Information Usefulness (IU) mediates the effect of information Quality (IQ) on Information Adoption (IA).

H4: Information Usefulness (IU) mediates the effect of Information Credibility (IC) on Information Adoption (IA).

H5: Perceived Bloggers’ Competence (PBC) positively moderates the indirect effect of Information Quality (IQ) on Information Adoption (IA) via Information Usefulness (IU).

H6: Perceived Bloggers’ Competence (PBC) positively moderates the indirect effect of Information Credibility (IC) on Information Adoption (IA) via Information Usefulness (IU).

## Materials and methods

### Research background

The data collection period of this study (October 3–20, 2025) coincided with a highly time-sensitive public health event: the influenza cluster outbreak in universities in Malaysia. According to local media reports, the outbreak caused widespread concern around October 13, causing some universities to suspend classes and issue health warnings to student groups through official mail [27]. This event provides a rare “historical moment” or natural experimental situation for this study. Although such immediate events still lack in-depth academic literature support, their real community communication background is to explore the role of college students in public health emergencies, social media and other channels provide an invaluable and timely window into how health information is accessed, assessed and responded to. This study considers this context as a key context and aims to capture the information behavior dynamics of individuals under real health risk perceptions [27].

### Participants

Based on the uniqueness and representatives of this group in the literature, Malaysian undergraduates were selected as the research sample. First, as early as 2003, a study showed that undergraduates are a high-practice group for self-care [9], which ensures a high correlation of between the study sample and the core phenomenon. Second, this group is an “aboriginal” in social media era, especially post-epidemic research has clearly pointed out that a large number of Malaysian undergraduates are very active in accessing information on social media (TikTok) [13], while this is not the case in Malaysia, which makes them observers social media. An ideal focal group that interacts with health information. In addition, choosing undergraduates with a relatively homogeneous educational background helps to control the “level of education”, a variable that has been proven to be related to self-perceived medical knowledge [12], so that the impact of other factors can be analyzed more accurately. Therefore, the selection of this sample effectively links the historical popularity of self-medication therapy, the contemporary use of social media, and the necessity of education level control.

This study was conducted in the context of the Malaysia’s health care system. The system is consists of public and private systems, which provide citizens with basic medical services of inclusive companies [7]. However, seeking public health services may face issues such as increased waiting times, which may lead some groups, especially young people who are familiar with digital technology, to seek public health services and turn to social media for initial health information and advice [28]. Despite the lack of authoritative national data on Malaysia’s self-medication rate, several regional studies have shown that young people obtain information through online channels for self-diagnosis and drug consultation [29]. It is in this context that this study aims to explore how young people active in social media evaluate and adopt online health information.

This study used a random sampling method to recruit undergraduate students from a University in Kuala Lumpur, Malaysia. First, we obtained the student list of the university through the student mailbox and compile it into a database. Then, used a computer-generated random number table to randomly select potential participants from the list to ensure that each student is selected equally. The recruitment period lasts from October 3 to October 23, 2025. The research team sent invitations to randomly selected students by email, including links to online surveys and detailed research information. All invitations clearly indicate that participation is completely voluntary, and the option of withdrawing from the study is provided. Participants are required to read and agree to an online informed consent form before starting the questionnaire. The table details the objectives, procedures, data confidentiality measures and potential impacts of the research. Only after obtaining consent can participants access and complete the online questionnaire. The online survey platform ensures anonymity and data security to encourage honest answers.

A total of 1,386 students answered and completed the online questionnaire. To ensure data quality and validity, we rigorously examined and screened the collected data according to the following criteria: (1) excluding questionnaires containing invalid personal information, such as incomplete or contradictory answers; (2) exclude questionnaires that contain invalid answers, such as too consistent answers or all questions answered with the same option. Ultimately, 1219 participants (648 male and 571 female) were included in the study. The mean age of participants was 20.47 years (SD = 1.67), with an age range of 18–23 years.

This study used an online questionnaire for probabilistic sampling, and while seeking to infer the population, we obtained a complete and up-to-date list of student mailboxes from the university registry as a sampling frame as far as possible, considering the sampling frame and coverage bias, to minimize the lack of coverage. At the same time, for non-response bias, we improved the response rate by multiple rounds of reminders (such as sending a reminder email). In the data processing stage, we compared the data characteristics of early respondents and late respondents (as late respondents may have more similar characteristics to non-respondents) to assess the possible impact of non-response bias. We also used the Wenjuanxing form link as a one-time visit link to prevent non-target group participation and repeated submission. Finally, to encourage the respondents to give true answers, we should avoid guiding language in the design of questions, we applied rigorous screening criteria in data analysis to eliminate invalid data and ensure the reliability of the results.

### Measures

To ensure the validity and reliability of the measurement tools in the target culture, all the scales in this study followed the systematic scale adaptation and validation process. First, all constructs were measured by the well-established English language scale, which is widely used in international journals and has been proven to have good reliability and validity. As the target participants of this study are undergraduate students from Malaysian universities, whose main language of teaching and academic communication is English, and they have strong English comprehension ability. In order to maximize the conceptual connotation of the original scale and avoid possible deviations in translation, this study directly uses the original English version of the questionnaire and tests the validity of the questionnaire with the questionnaire.

On this basis, to ensure the applicability of the scale in the specific research context of social media health information and the Malaysian cultural context, we took the following steps for cultural validation and content validity improvement. And three experts in the field of health communication and social media (two professors and one professor’s doctoral student) were invited to review the adjusted scale. Experts provided feedback on the clarity of the project presentation, the relevance to the research context and the cultural suitability. According to the opinion of experts, the wording of some projects has been optimized to improve their clarity. Before the formal investigation, we conducted a small pilot test (N = 30, from the target group, but did not participate in the formal study) [30]. The purpose of the pretest was to assess the overall understanding of the questionnaire, the completion time and whether there was any ambiguity in the item statements. Participant feedback showed that all scale items were easy to understand and no significant cultural or semantic confusion was reported. The preliminary reliability coefficients (Cronbach’s α) based on the pretest data were all above 0.8, indicating that the scale had good internal consistency.

All scales were conducted in English, and English is the main language of the target participants, thus avoiding potential deviations related to translation and ensuring the conceptual consistency of key variable measurements, such as information quality, information usefulness and information adoption. This method enhances the effectiveness and cross-context comparability of structural measurement..

#### Information Quality (IQ).

The Information Quality Scale was used to evaluate the quality of health-related information on social media [19,18]. The scale consists of 7 items, such as “Public health information about the recent flu outbreak on TikTok is accurate.” It is scored on a 5-point Likert scale, ranging from 1 (strongly disagree) to 5 (strongly agree). Higher total scores indicate a higher perceived quality of health information. In this study, the Cronbach’s α coefficient for this scale was 0.903.

#### Information Credibility (IC).

The Information Credibility Scale [8,31] was adapted to measure the trustworthiness of health information sources. The scale consists of 3 items: “ Public health information about the recent flu outbreak on TikTok is trustworthy.” It is scored on a 5-point Likert scale, ranging from 1 (strongly disagree) to 5 (strongly agree). Higher total scores indicate a more substantial perceived credibility of information sources. In this study, the Cronbach’s α coefficient for this scale was 0.838.

#### Information Usefulness (IU).

Information Usefulness. The Perceived Usefulness Scale [24,32] was applied to assess the practical value of health information. The scale consists of 3 items: “ Public health information about the recent flu outbreak on TikTok is Useful for health decisions.” It is scored on a 5-point Likert scale, ranging from 1 (strongly disagree) to 5 (strongly agree). Higher total scores indicate greater perceived usefulness. In this study, the Cronbach’s α coefficient for this scale was 0.850.

#### Information Adoption (IA).

Information Adoption. The Information Adoption Scale [13,33] measured behavioral intentions toward health information. The scale consists of 3 items: “I plan to make use of public health information about the recent flu outbreak on TikTok. “It is scored on a 5-point Likert scale, ranging from 1 (strongly disagree) to 5 (strongly agree). Higher total scores indicate stronger adoption intentions. In this study, the Cronbach’s α coefficient for this scale was 0.837.

#### Perceived Bloggers’ Competence (PBC).

Perceived Bloggers’ Competence. The perceived competence of health information providers on TikTok was measured using an adapted scale [34]. The measurement was contextualized for three distinct source types: Official Medical Institutions/Certified Health Experts/Doctors/Lifestyle Bloggers (e.g., “Please evaluate your perception of the competence of the official medical institution when viewing influenza information posted by them on TikTok: ‘This publisher has professional knowledge.’). All items were rated on a 5-point Likert scale from 1 (strongly disagree) to 5 (strongly agree). Higher scores indicate greater perceived competence. The scale demonstrated high reliability in this study (Cronbach’s α = 0.901).

### Statistical analyses

This study used SPSS 27.0 and Amos 27.0 to analyze the data. First, to assess common method bias, Harman single-factor test was performed, and Pearson correlation coefficients between descriptive statistics and variables were calculated to preliminarily investigate their relationships. The first stage focused on the validation of the measurement model, we assessed the internal consistency reliability of the scale by calculating Cronbach’s α coefficient [35], and utilized Amos to perform CFA/SEM to test the construct validity of the measurement model. By reporting model fit indices (such as Χ2/DF, CFI, TLI, RMSEA, SRMR) and calculating combined reliability with mean variance extraction values [36], it is ensured that all variables are effectively measured by the corresponding observed variables, laying a reliable foundation for subsequent hypothesis testing. The second stage focused on hypothesis testing of structural models; we tested research hypotheses using SPSS PROCESS. The simple mediating effect (H1-H4) was tested by Model 4, and the moderated mediating effect (H5-H6) was tested by Model 7 [29]. All analyses used the 5000-bootstrap method, and the 95% confidence interval without zero was considered significant [37].

### Ethical considerations

This study was conducted in strict compliance with the ethical principles of the Declaration of Helsinki regarding medical research involving human subjects. All research procedures were approved by the Research Ethics Committee of the University of Putra Malaysia (UPM) on 16 September 2023(approval number: JKEUPM-2023–823). We ensured that the core principles of the declaration were implemented throughout the research process; These include: voluntary participation (all participation is based on fully informed consent), benefit and risk minimization (the study was designed as an anonymous questionnaire and involved only minimal risk), privacy and confidentiality (data collection and processing were conducted anonymously and in accordance with the university’s data protection guidelines), and subject rights (participants have the right to withdraw from the study at any time without reason).

To ensure participant privacy, this study was conducted anonymously in full compliance with UPM’s guidelines for data protection in international research collaborations. Before the start of data collection, all potential participants gave informed consent in English about the detailed purpose of the study, procedures, potential benefits and risks, data confidentiality measures, and outcomes, and their right to withdraw at any time. Access to and completion of the formal questionnaire will only be granted if the “I agree” option is explicitly clicked on the electronic informed consent page.

All participants in this study were aged 18–23 years. In accordance with Malaysian law and internationally accepted ethical guidelines for research, an adult who has attained the age of 18 is considered to have full civil capacity and has the legal right to independently provide informed consent [38]. In reviewing the study protocol, this ethics committee assessed and concluded that this study was in the lowest risk category, the content of the study (social media health information behavior) is highly related to the participants’ daily life experience, which is easy to understand. Therefore, the ethics committee determined that all participants 18 years of age and older had adequate capacity to interpret research information and make autonomous participation decisions. Based on this, the committee approved the process of obtaining informed consent for this study only from the participants themselves, waiving the requirement to obtain parental or guardian consent. All adult participants, regardless of age, followed the uniform informed consent procedure described above.

## Results

### Descriptive statistics

Table 1 presents the means, standard deviations, and Pearson correlations among the five focal variables for the entire sample (N = 1219). Respondents reported moderate-to-high levels of Information Quality (IQ; M = 3.105, SD = 0.745), Information Credibility (IC; M = 3.071, SD = 0.891), Information Usefulness (IU; M = 3.006, SD = 0.902), and Perceived Bloggers’ Competence (PBC; M = 3.39, SD = 1.08), whereas actual Information Adoption (IA) was somewhat lower on average (M = 3.317, SD = 1.092).

**Table 1:** Means, Standard Deviations, and Correlation Matrix of Key Variables (N = 1219).

Variables	M	SD	1	2	3	4	5
1. IQ	3.105	0.745	1				
2. IC	3.071	0.891	0.164**	1			
3. IU	3.006	0.902	0.321**	0.235**	1		
4. IA	3.017	0.853	0.313**	0.281**	0.495**	1	
5. PBC	3.317	1.092	0.031	−0.021	−0.087**	−0.027	1

Notes: **p < 0.001; IQ = Information Quality, IC = Information Credibility, IU = Information Usefulness, IA = Information Adoption, PBC = Perceived Bloggers’ Competence.

The results of descriptive statistics and correlation analysis are shown in Table 1. The core variables of Information Quality (IQ), Information Credibility (IC), Information Usefulness (IU) and Information Adoption (IA) were significantly positively correlated with each other, especially the correlation between Information Usefulness (IU) and Information Adoption (IA) was the strongest (r = 0.495, p < 0.001), which provided preliminary support for the research model. As a moderator variable, Perceived Bloggers’ Competence (PBC) has no significant correlation with other variables and is not correlated with the dependent variable, indicating that the moderator variable and the main effect variable are not endogenous and in an ideal exogenous state. These descriptive statistics and inter-correlations provide initial support for our mediation and moderation hypotheses and justify further testing of the proposed structural model.

### Reliability and validity analysis

#### Internal validity.

In order to improve the internal validity of the sample data, we carried out a strict data cleansing process: through the platform Cookie and IP address, we screened and eliminated the duplicate questionnaires; The short time (less than 2/3 of the normal time) for completing the questionnaire was eliminated, and the samples with wrong answers in the attention test items were excluded Responses that showed significant deviations from the regular response pattern were removed.

#### Convergent validity.

As shown in Table 2, In terms of validity analysis, the Kaiser-Meyer-Olkin (KMO) measure of sampling adequacy and Bartlett’s test of spheric were first conducted to assess the suitability of the data for factor analysis. The results showed a KMO value of 0.866, and Bartlett’s test of spheric reached a significant level (χ² = 12311.820, df = 171, p < 0.001). This indicates the presence of significant underlying structures among the variables, confirming that the data are highly suitable for factor analysis and exhibit good construct validity. Also, A confirmatory factor analysis (CFA) was conducted to assess the measurement model. To check for common method bias, Harman’s single-factor test was performed. The results showed that the first factor accounted for 29.882% of the total variance (eigenvalue = 5.678), which is below the critical threshold of 50%, indicating that common method bias is not a serious concern in this study. The CFA results demonstrated that all factor loadings of the measurement items exceeded the acceptable standard of 0.70, ranging from 0.762 to 0.941, confirming that each indicator effectively represents its corresponding latent variable. Regarding reliability, the values for Cronbach’s alpha for all constructs were above 0.80, and the composite reliability (CR) values, ranging from 0.861 to 0.937, surpassed the recommended value of 0.70. For convergent validity, the average variance extracted (AVE) for each construct exceeded the 0.50 benchmark, with values between 0.613 and 0.833. In summary, the measurement model demonstrates adequate reliability and validity, supporting the robustness of the subsequent analyses.

**Table 2:** Convergent validity (N = 1219).

Constructs’ Indicators	Items	Factor Loadings	Cronbach’s alpha	Composite reliability (CR)	Average variance extracted (AVE)
Information Quality (IQ)	IQ1: Public health information about the recent flu outbreak on TikTok is accurate.	0.763	0.903	0.917	0.613
	IQ2: Public health information about the recent flu outbreak on TikTok is believable.	0.791			
	IQ3: Public health information about the recent flu outbreak on TikTok is clear.	0.792			
	IQ4: Public health information about the recent flu outbreak on TikTok is coherent.	0.762			
	IQ5: Public health information about the recent flu outbreak on TikTok is comprehensive	0.790			
	IQ6: Public health information about the recent flu outbreak on TikTok is concise.	0.796			
	IQ7: Public health information about the recent flu outbreak on TikTok is well-written.	0.787			
Information Credibility (IC)	IC1: Public health information about the recent flu outbreak on TikTok is important.	0.855	0.838	0.928	0.808
	IC2: Public health information about the recent flu outbreak on TikTok is factuality.	0.854			
	IC3: Public health information about the recent flu outbreak on TikTok is trustworthiness.	0.858			
Information Usefulness (IU)	IU1: Public health information about the recent flu outbreak on TikTok is Useful for health decisions.	0.845	0.850	0.870	0.691
	IU2: Public health information about the recent flu outbreak on TikTok is Improved understanding of health issues.	0.827			
	IU3: Public health information about the recent flu outbreak on TikTok is actionable guidance.	0.821			
Information Adoption (IA)	IA1: I plan to make use of public health information about the recent flu outbreak on TikTok.	0.825	0.837	0.861	0.674
	IA2: I will frequently adopt public health information about the recent flu outbreak on TikTok.	0.807			
	IA3: I will recommend that others use public health information about the recent flu outbreak on TikTok.	0.830			
Perceived Bloggers’ Competence (PBC)	PBC1: Please evaluate your perception of the competence of the official medical institution when viewing influenza information posted by them on TikTok: ‘This publisher has professional knowledge.’	0.857	0.901	0.937	0.833
	PBC2: Please evaluate your perception of the competence of the certified health expert/doctor when viewing influenza information posted by them on TikTok: ‘The information provided by this publisher is supported by scientific evidence.’	0.941			
	PBC3: Please evaluate your perception of the competence of the lifestyle blogger you follow when viewing influenza information posted by them on TikTok: ‘This publisher has relevant qualifications.’	0.938			

#### Discriminant validity.

The Fornell-Larcker criterion was applied to evaluate discriminant validity. As shown in Table 3, the square roots of AVE values (diagonal bolded values) for all constructs exceeded the off-diagonal correlations with other constructs, confirming distinctiveness between latent variables. This demonstrates that each construct shares more variance with its indicators than others, fulfilling discriminant validity requirements.

**Table 3:** Fornell-Larcker Criterion for Discriminant Validity (N = 1219).

Construct	IQ	IC	IU	IA	PBC
IQ	**0.783**				
IC	0.164	**0.899**			
IU	0.321	0.235	**0.831**		
IA	0.313	0.281	0.495	**0.821**	
PBC	0.031	−0.021	−0.087	−0.027	**0.913**

Notes: IQ = Information Quality, IC = Information Credibility, IU = Information Usefulness, IA = Information Adoption, PBC = Perceived Bloggers’ Competence.

### Confirmatory factor analysis

**Fig 2:**
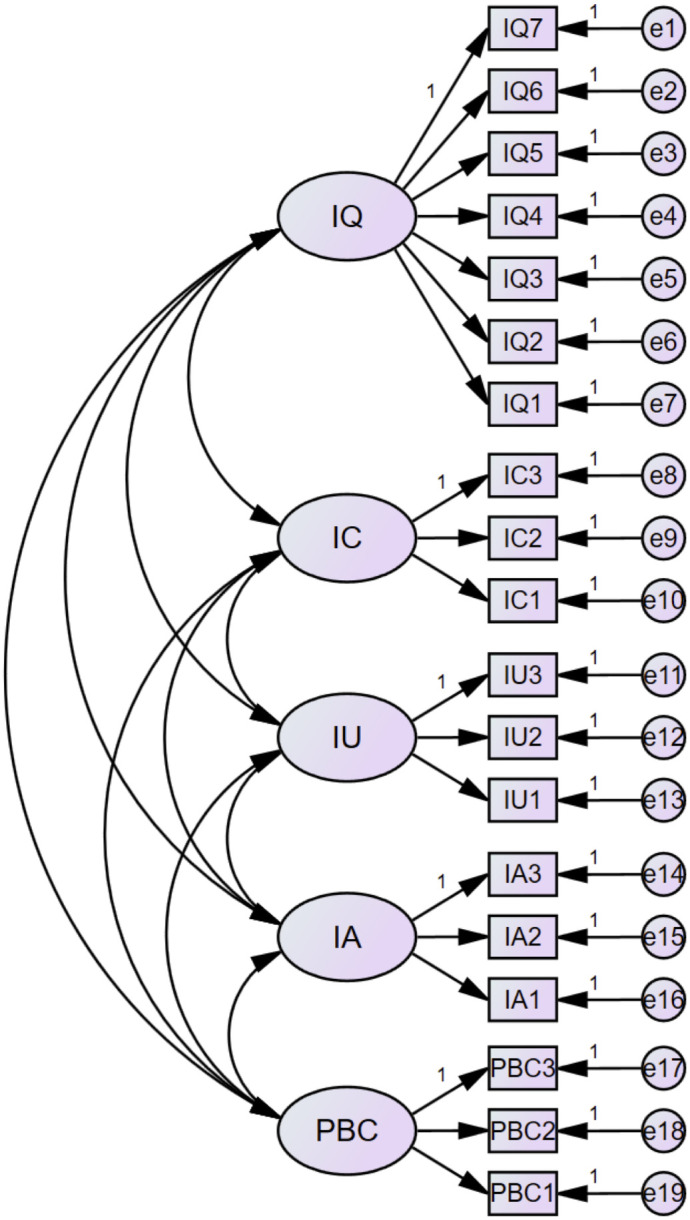
Structural equation modeling path (N = 1219). Notes: IQ = Information Quality, IC = Information Credibility, IU = Information Usefulness, IA = Information Adoption, PBC = Perceived Bloggers’ Competence.

The structural model demonstrated an excellent fit to the data. The ratio of chi-square to degrees of freedom (CMIN/DF = 1.621) falls well below the commonly accepted threshold of 3.0, indicating minimal discrepancy between the hypothesized model and the observed covariance matrix. Incremental fit indices were uniformly strong: the Incremental Fit Index (IFI = 0.993), Tucker–Lewis Index (TLI = 0.991), and Comparative Fit Index (CFI = 0.993) all exceed the more stringent criterion of 0.95, reflecting superior relative improvement over a null model. Residuals were likewise minimal, with a Root Mean Square Residual (RMR = 0.017) below the recommended maximum of 0.05 and a Root Mean Square Error of Approximation (RMSEA = 0.023) well under the 0.06 cutoff for good fit (90% CI not shown). These indices provide robust evidence that the proposed mediated‑moderation model accurately captures the relationships among information quality, source credibility, perceived usefulness, blogger competence, and behavioral adoption. The measurement model demonstrated strong convergent validity and reliability across all four latent constructs. The results are shown in Fig 2:

### Standardized bootstrapped mediation effect

The results of the mediation analysis (Model 4) are presented in Table 4. The model examined the relationship between Information Quality (IQ), Information Usefulness (IU), and Information Adoption (IA). First, the path from Information Quality to Information Usefulness (the IQ → IU relationship) was found to be positive and statistically significant (*β* = 0.389, p < 0.001), thus providing support for H1.

**Table 4:** Direct and Indirect Effects of Information Quality on Information Adoption through Information Usefulness.

Path	Effect Type	Coeff.	SE	t	p	Bootstrap 95% CI
**Direct Effect**						
IQ → IA	Direct Effect	0.196	0.030	6.632	<.001	[0.138, 0.255]
**Indirect Effect Path**						
IQ → IU	(Path a)	0.389	0.033	11.832	<.001	–
IU → IA	(Path b)	0.416	0.024	17.005	<.001	–
IQ → IU → IA	Indirect Effect	0.162	0.017	–	–	[0.129, 0.196]

Notes: N = 1219. The model for the mediator (Information Usefulness) accounted for a significant portion of variance, *R²* = 0.103, *p* < 0.001. The full model for the outcome (Information Adoption) also showed a good fit, *R²* = .271, *p* < .001. Bootstrap sample size = 5000.IQ = Information Quality, IU = Information Usefulness, IA = Information Adoption.

Furthermore, the analysis reveals a significant indirect effect of Information Quality on Information Adoption through Information Usefulness, as evidenced by a bootstrap confidence interval that does not include zero (*β* = 0.162, Boot SE = 0.017, 95% Boot CI [0.129, 0.196]). This indicates that information usefulness serves as a partial mediator in the relationship between Information Quality and Information Adoption. Therefore, H3, which proposed this mediating effect, is supported.

The results of the mediation analysis are presented in Table 5. The path from Information Credibility (IC) to Information Usefulness (IU) was positive and statistically significant (*β* = 0.238, *p* < 0.001), providing support for the corresponding hypothesis (H2).

**Table 5:** Direct and Indirect Effects of Information Credibility on Information Adoption through Information Usefulness (N = 1219).

Path	Effect Type	Coeff.	SE	t	p	Bootstrap 95% CI
**Direct Effect**						
IC → IA	Direct Effect	0.167	0.024	6.940	<.001	[0.120, 0.214]
**Indirect Effect Path**						
IC → IU	(Path a)	0.238	0.028	8.446	<.001	–
IU → IA	(Path b)	0.429	0.024	18.038	<.001	–
**IC → IU → IA**	**Indirect Effect**	**0.102**	**0.015**	–	–	**[0.074, 0.133]**

Note: The model for the mediator (Information Usefulness) was significant, *R²* = 0.055, *p* < 0.001. The full model for the outcome (Information Adoption) was also significant, *R²* = 0.274, *p* < 0.001. Bootstrap sample size = 5000. IC = Information credibility, IU = Information Usefulness, IA = Information Adoption.

Moreover, the analysis revealed a significant indirect effect of information credibility on information adoption through Information Usefulness, as the bootstrap confidence interval did not include zero (*β* = 0.102, Boot SE = 0.015, 95% Boot CI [0.074, 0.133]). This indicates that Information Usefulness acts as a significant mediator in the relationship. Therefore, the hypothesis proposing this mediation (H4) is supported.

### Moderation hypotheses analysis

The analysis confirms a significant moderated mediation effect. As shown in Table 6 Part A, the interaction between Information Quality (IQ) and Perceived Bloggers’ Competence (PBC) on Information Usefulness (IU) is significant (*β* = −0.203, *p* < 0.001). Table 6 Part B shows the effect of IQ on IU is strongest when PBC is low (*β* = 0.631, *p* < 0.001), and weakest when PBC is high (*β* = 0.158, *p* < 0.001). Consequently, the indirect effect of IQ on Information Adoption through IU is also moderated, ranging from 0.262 (low PBC) to 0.066 (high PBC). Based on the results in Table 6, the analysis reveals a significant negative moderating effect of Perceived Blogger Competence (Effect = −0.084, 95% CI [−0.110, −0.059]), which contradicts a hypothesis (H5) predicting a positive effect.

**Table 6:** Results of the Moderated Mediation Analysis (Information Quality). Model Summary: The model examined whether the indirect effect of Information Quality on Information Adoption through Information Usefulness is moderated by Perceived Bloggers’ Competence. All variables were mean centered prior to analysis. N = 1219. Bootstrap sample size = 5000.

Part A: Regression Model for the Mediator (Information Usefulness, IU)				
Predictor	Coeff.	SE	t	p
Constant	3.011	0.024	125.956	<0.001
Information Quality (IQ)	0.364	0.032	11.255	< 0.001
Perceived Bloggers’ Competence (PBC)	−0.078	0.022	−3.568	< 0.001
Interaction (IQ x PBC)	−0.203	0.029	7.029	< 0.001
Model Fit	R² = .147	MSE = .696	F (3, 1215) = 69.910	p < 0.001
**Part B: Conditional Effects of Information Quality on Information Usefulness at Values of the Moderator (W)**								
**Level of PBC (W)**	**Effect**	**SE**	**t**	**p**	**95% CI**	**Indirect Effect**	**BootSE**	**95% BootCI**
Low (−1.3169)	0.631	0.047	13.512	< 0.001	[.540,.723]	0.262	0.021	[0.220, 0.305]
Mean (0.0164)	0.361	0.032	11.131	< 0.001	[.297,.425]	0.150	0.017	[0.118, 0.185]
High (1.0164)	0.158	0.046	3.415	< 0.001	[.067,.249]	0.066	0.024	[0.021, 0.115]
Index of Moderated Mediation	–	–	–	–	–	−0.084	0.013	[-0.110, -0.059]

Note: IQ = Information Quality, IU = Information Usefulness, IA = Information Adoption, PBC = Perceived Blogger Competence. Variables were mean-centered. N = 1219. Bootstrap samples = 5000.

The results of the moderated mediation analysis are presented in Table 7. As shown in Part A, the interaction between Information Credibility and Perceived Bloggers’ Competence on Information Usefulness is significant and negative (*β* = −0.309, p < 0.001). Part B clarifies this effect, showing that the positive effect of Information Credibility on Information Usefulness is strongest when Perceived Bloggers’ Competence is low (*β* = 0.675, p < .001), but diminishes and becomes non-significant when Perceived Bloggers’ Competence is high (*β* = −0.045, *p* = 0.192). Consequently, the corresponding indirect effect on Information Adoption is also strongest at low competence levels (Effect = 0.290) and non-significant at high levels (Effect = −0.019). This pattern of a weakening effect is confirmed by the significant negative Index of Moderated Mediation (Effect = −0.132, 95% CI [−0.156, −0.109]). Therefore, the results demonstrate a significant negative moderating effect, which is contrary to the hypothesized positive effect (H6). This suggests that a blogger’s high competence may act as a heuristic cue, reducing users’ reliance on evaluating the credibility of the information.

**Table 7:** Results of the Moderated Mediation Analysis (Information Credibility). Model Summary: The model examined whether the indirect effect of Information Credibility on Information Adoption through Information Usefulness is moderated by Perceived Bloggers’ Competence. All variables were mean centered prior to analysis. N = 1219. Bootstrap sample size = 5000.

Part A: Regression Model for the Mediator (Information Usefulness, IU)				
Predictor	Coeff.	SE	t	p
Constant	3.000	0.024	127.520	< 0.001
Information Credibility (IC)	0.269	0.027	10.140	< 0.001
Perceived Bloggers’ Competence (PBC)	−0.062	0.022	−2.860	0.004
Interaction (IC x PBC)	−0.309	0.024	−12.821	< 0.001
Model Fit	R² = .147	MSE = .674	F (3, 1215) = 69.910	< 0.001
**Part B: Conditional Effects of Information Credibility on Information Usefulness at Values of the Moderator (W)**								
**Level of PBC (W)**	**Effect**	**SE**	**t**	**p**	**95% CI**	**Indirect Effect**	**BootSE**	**95% BootCI**
Low (−1.3169)	0.675	0.043	15.623	< 0.001	[0.591, 0.760]	0.290	0.022	[0.248, 0.332]
Mean (0.0164)	0.264	0.027	9.962	< 0.001	[0.212, 0.316]	0.113	0.013	[0.089, 0.140]
High (1.0164)	−0.045	0.034	−1.304	0.192	[-0.112, 0.023]	−0.019	0.017	[-0.052, 0.014]
Index of Moderated Mediation	–	–	–	–	–	−0.132	0.012	[-0.156, -0.109]

Note: IC = Information Credibility, IU = Information Usefulness, IA = Information Adoption, PBC = Perceived Bloggers’ Competence. Variables were mean-centered. N = 1219. Bootstrap samples = 5000.

## Discussion

The findings of this study clarified the complex interplay between Perceived Bloggers’ Competence (PBC), Information Quality (IQ), Information Credibility (IC), and Information Usefulness (IU), drawing on Social Cognitive Theory (SCT) and the Information Adoption Model (IAM). By integrating dynamic trust mechanisms and platform-specific contextualization, this study extended previous theoretical framework and provided new insights into the cognitive and social processes behind digital health communication [39].

The research results are consistent with the principles of Social Cognitive Theory (SCT) and Information Adoption Model (IAM), which provides strong support for the basic hypothesis on the impact of information characteristics. Specifically, both information quality (IQ) and information credibility (IC) significantly improve the usefulness (IU) of public health information (support H1 and H2, respectively), emphasizing the role of information usefulness as a core cognitive mechanism for individual assessment and operational health information. These findings are consistent with previous research results, indicating that users will actively evaluate the substance quality and source reliability of the information before adopting it [40] and extend that work by showing usefulness serves as a key evaluative filter.

Building on Bandura’s concept of observational learning [22], the study further explores how Perceived Bloggers’ Competence (PBC), as a social cue shapes these cognitive paths. Through moderated mediation analysis, this study reveals a key boundary condition of perceived blogger competence (PBC) in the process of information adoption. Unlike the originally assumed prediction direction, PBC has a significant negative adjustment effect on the two intermediary paths. Although it is assumed that higher blogger ability (H5 and H6) will enhance this relationship, the data shows that the opposite is true: specifically, when the PBC is lower, this relationship is stronger, and the information content (quality and credibility) has a stronger impact on adoption through usefulness; this effect is significantly weaker or even non-significant when PBC is high [38]. The reliability of the present study measurement tool is good, and Cronbach’s alpha of all constructs is greater than 0.80 and composite reliability values ranges from 0.837 to 0.903, which meet psychometric standard [41]. The study found that when bloggers are considered more capable, the positive impact of information quality and credibility on information usefulness will be weakened. This discovery has robustness and exploratory value. This consistent finding prompts us to go beyond hypothesis testing and re-interpret the complex role of PBC from the theoretical perspective of “competence discounting”.

The “competence discounting” mechanism originates from SCT and IAM [42], which means that when the information source is considered highly reliable in the ability dimension, the audience will reduce its continuous dependence on the ability clue, and when the information source is considered to be highly reliable, the audience’s cognitive resources may be transferred. To other evaluation channels, or they may directly rely on inspirational clues to make judgments [16]. In the context of the present study, this provides a central explanation for the negative regulation of PBC: as a powerful peripheral heuristic clue, high-ability perception may trigger the user’s heuristic processing mode, and the impact of high-ability perception on PBC performance is not obvious, making it dependent on the “expert” label. The psychological shortcut reduces the systematic evaluation of the content of the information itself, leading to a “discount” on the impact of the content path [16]. On the contrary, low perception forces the audience to conduct a deeper content assessment and amplifies the impact of information quality and credibility [43].

In the high-risk and high-uncertainty situation of public health crisis, this effect is further strengthened. First, information overload encourages users to reduce their assessment of information usefulness and to relying on source heuristics [42]. Secondly, extremely high professional expression may stimulate psychological resistance [43], causing users to subconsciously belittle the value of information in order to maintain decision-making autonomy. Third, high-competence information may reduce perceived applicability by being too technical [44]. Finally, the public’s emotional need for certainty may rapidly shift it from assessment information to authority, beyond rational content assessment [16].

More critically, this study reveals how this competence discounting effect is systematically moderated by platform characteristics. In short, the depth and way of users’ information processing is the result of the interaction of blogger image and platform characteristics. On the one hand, when bloggers appear unprofessional, the quality of information itself becomes crucial, which is consistent with the search for compensatory information [15], that is, when there is a lack of reliable source clues, the information quality itself becomes critical, and users turn to make a more in-depth assessment of the content. On the other hand, if bloggers are regarded as experts, they may trigger an automatic trust mechanism [15], and users perceive their professionalism as a heuristic cue, thus reducing cognitive effort. This study found that the intensity of this effect varies between different platforms. The visual presentation of the video platform can more effectively convey non-verbal trust clues and professional models, indicating that the visual presentation of the video platform is more effective in conveying non-verbal trust clues and expert models. Therefore, the ability discount effect may be Triggered earlier and more strongly, which echoes the richness of media and the literature established by trust [14], highlights the key role of platform media attributes in shaping initial trust perceptions [45].

These findings are of far-reaching practical significance to the spread of public health. The observed “competence discounting” effect reveals a key challenge: While expert sources are critical to building credibility, their highly perceived expertise can unintentionally undermine the public’s systematic handling of health information.. In infectious disease control or vaccination campaigns, if the public relies only on “expert” heuristics and ignores an in-depth understanding of the information content, it may lead to compliance with prevention and control guidelines based on fragile trust rather than solid understanding. Therefore, effective health communication must balance professional authority and cognitive accessibility and ensure that authoritative information is presented in a way that promotes (not replaces) public cognitive participation. Health authorities and communicators should strive to transform complex information into practical guidance, presenting the content itself in a transparent and empathetic manner, while conveying professional images with the help of rich media such as videos, so as to bridge the gap between expert knowledge and public understanding in the crisis.

By introducing and verifying the “competence discounting” mechanism, this study reveals the dynamic and sometimes contradictory interaction between source credibility and content quality in online health information adoption. This not only calls for the establishment of a more refined theoretical model, combining cognitive mechanisms with situational adjustment factors, which provides a new perspective for online health information adoption and research, but also provides an important strategic inspiration for effective public health communication on multimedia platforms: when building a professional image, the content presentation must be carefully designed., to activate rather than bypass the deep processing of the public; professional image construction must also be carefully designed to activate rather than bypass the deep processing of the public, so as to achieve the dual goal of trust and understanding.

## Implications

The findings of this study provide important theoretical and practical implications for public health communicators, social media platforms, and content creators. Practically, the research results highlight the need to develop an integrated communication strategy, that is, to seek a balance between technical expertise and audience participation. Bloggers should give priority to displaying dynamic proof of ability – such as real-time rumor refutation, interactive Q&A sessions or simplified data visualization – because these dynamic displays can enhance audience trust more effectively than static credentials [46]. For example, integrating visual tools to popularize complex medical information can significantly improve the audience’s understanding and willingness to adopt it; this is especially noticeable on visual-oriented platforms such as Instagram, because on such platforms, non-verbal clues can amplify the audience’s perception of the communicator’s ability [47]. However, if the high complexity of technology is overemphasized, it may trigger a “competence discounting”, that is, high professional competence can, paradoxically, undermine the trust of audiences by reducing the perceived accessibility of information. To alleviate this problem, bloggers should adopt a hierarchical communication strategy, a tailor-made communication method that aims to design the core focus and presentation style of information according to the audience’s information processing mode or perception of the ability of information sources. In the specific implementation, concise and easy-to-understand abstracts can be combined with selected links pointing to in-depth technical content.

In addition, public health institutions and online platforms should work together to improve the quality of information. For example, relevant systems could be introduced to automatically mark authoritative content, and algorithms can be used to warn and identify false information [29]. In the process of information dissemination, emotional content containing personal health stories should be cleverly integrated. Because compared with the boring list of facts, storytelling can attract the audience more and stimulate their motivation to turn knowledge into practical action, thus bridging the “competence discounting” [15]. According to the characteristics of different platforms, differentiated communication strategies need to be adopted: for example, text-oriented platforms such as X (formerly Twitter) are suitable for publishing structured interpretation articles to clarify technical terms; while on short video platforms such as TikTok, experts can be invited to launch “rumor challenges” and other mutual Active activities. In addition, it is also an effective strategy to set up a quick response team to clarify misunderstandings and correct misinformation in the blogger’s comment area [16].

Theoretically, this study contributes to the integration of Social Cognitive Theory (SCT) and the Information Adoption Model (IAM), addressing key gaps in health communication research. First, this study empirically demonstrates the key role played by the trust mechanism (such as emotional resonance and interactive participation) in driving information adoption, thus challenging the theoretical orientation dominated by rationalism in the IAM model. These research findings highlight the emphasis of social cognitive theory (SCT) on observational learning and social impact [22], revealing that trust is not only evaluated through cognition, but is jointly constructed at the social level through interactive clues. Secondly, this study reconceptualizes information credibility into a dynamic and exhibiting attribute, showing that compared with formal credentials, the perceived bloggers’ competence can be more effective through real-time interaction. [16]. Third, the research introduces the context factors of a specific platform into the theory of health communication, focusing on how the visual platform strengthens the regulatory role of bloggers’ perception ability through non-verbal trust clues – this discovery coincides with the emerging neurocognitive model of value calculation in the current media environment. [20]. The most inspiring discovery in this study lies in the complex role played by the perception bloggers’ competence. Contrary to the expectations based on the assumptions of H5 and H6, empirical results show that perception bloggers’ competence (PBC) has produced significant results in the two intermediary paths of “information quality → information usefulness → information adoption” and “information credibility → information usefulness → information adoption”. In other words, the higher the audience’s perception of the blogger’s competence, the indirect positive impact of information quality and information credibility on information adoption through the intermediary variable of information usefulness will be weakened. This unexpected discovery reveals a “Competence discounting” effect worthy of in-depth exploration; this discovery is of important theoretical value for understanding the dynamics of trust and its cognitive decision-making mechanism in the context of digital health communication.

## Limitations

When interpreting the findings of this study, some of its limitations should be fully considered. First of all, the measurement of core variables in this study (such as health information adoption behavior and perception of bloggers’ competence) depends entirely on the questionnaire survey data. Although we have adopted methods such as the Harman single-factor test to assess the risk of common method bias, social approval bias and recall bias may still have an impact on the reliability of data.

Secondly, this study adopts a cross-sectional design, so the causal relationship between variables cannot be established; future research needs to adopt longitudinal design or experimental design to verify the causal path between variables. In addition, the research situation of this study has specific platform and cultural attributes. The data is collected from a specific social media platform (TikTok) and a specific cultural background (Malaysia), and potential influencing factors such as algorithm content recommendations, cultural attitudes towards authority, and differences in digital health literacy are not considered. Therefore, it is necessary to be cautious when extending the conclusions of this study to other platforms or cultural situations.

In addition, although this study examines the perception blogger’s competence as a moderation variable, it does not take into account potential situational variables such as algorithm content curation, cultural attitude towards authority, and differences in digital health literacy – and the above factors may affect information processing and adoption behavior, thus producing the “life shaping” effect [48].

Finally, the unexpected negative moderation effect of perceived blogger competence invites further exploration. This result may be due to the pseudonym in the measurement process (such as the “ceiling effect” in the ability assessment), or it may reflect a deeper psychological mechanism – such as the audience’s skepticism towards those highly authoritative or overly modified information sources. Future research should consider further improving the operational definition of the concept of “competence” and explore other potential explanations based on the theory of trust dynamics and heuristic processing.

Future research can use experiments to directly manipulate perceived bloggers’ competence to test the above theoretical mechanism, it can also be combined with longitudinal tracking research to explore the boundary effect of different cultural backgrounds and health issue risk levels on the “competence discounting” effect, so as to reveal the sense of ability. The dynamic evolution law of the relationship between knowledge and information adoption. By deepening and expanding at the theoretical level and closely combining with practical application, this study not only provides a new solution for the response to the phenomenon of “competence discounting” in the field of digital health communication, but also lays a solid theoretical foundation for building a more efficient health communication strategy.

## Conclusion

Findings from this study provide strong empirical support for all proposed hypotheses. Both information quality and information credibility have a significant positive impact on information usefulness, which in turn fully mediates their effects on health information Adoption (*β* = 0.162 and 0.102, respectively; p < .001), extending [29] the Information Adoption Model to the field of health communication based on social media. However, contrary to conventional expectations, perceived bloggers’ competence negatively moderates the relationship between information usefulness and information adoption (Effect = −0.084, 95% CI [−.110, −.059]; Effect = −0.132, 95% CI [−.156, −.109]), suggesting a “competence discounting” effect. This paradox shows that although ability can enhance trust in some situations, under certain conditions such as excessive cognitive load, excessive professionalism may weaken the audience’s independent perception or cause heuristic disengagement.

By integrating Social Cognitive Theory (SCT), the study addresses Information Adoption Model’s rationalist limitations, and emphasizes that trust does not only come from rational cognitive evaluation, but is jointly constructed through emotional interaction and situational interaction. The results confirmed that the “interactive credibility signal” was more relevant than the “static credentials”. Health communicators should adopt a hierarchical communication strategy to seek a balance between authority and affinity, and combine content with real-time and emotional resonance, so as to bridge the “competence discounting” [22] knowledge-action gap. Although this study helps to deepen the detailed understanding of the adoption of health information in digital contexts, its limitations cannot be ignored. The cross-sectional research design limits the inference of causality. Future research should adopt longitudinal or experimental approaches to track how perceived bloggers’ competence change over time, and explore how these perceptions interact with cultural, algorithmic, and contextual variables across platforms.

In summary, this study has built a hybrid theoretical framework that integrates IAM and SCT, which can capture the dual dimensions of digital trust at the cognitive and social emotional levels at the same time. By revealing how perceived bloggers’ competence dynamically moderate information adoption pathways, this study provides insights and suggestions with practical value for public health stakeholders who are committed to optimizing online communication strategies in an increasingly complex media ecosystem.
